# Immune Dysfunction and Albumin-Related Immunity in Liver Cirrhosis

**DOI:** 10.1155/2019/7537649

**Published:** 2019-02-25

**Authors:** Benjamin Wilde, Antonios Katsounas

**Affiliations:** ^1^Department of Nephrology, University Hospital Essen, University Duisburg Essen, Hufelandstrasse 55, 45122 Essen, Germany; ^2^Department of Gastroenterology, Hepatology and Infectious Diseases, University Magdeburg, Leipzigerstrasse 44, 39120 Magdeburg, Germany

## Abstract

Liver cirrhosis yearly causes 1.2 million deaths worldwide, ranking as the 10th leading cause of death in the most developed countries. High susceptibility to infections along with a significant risk for infection-related mortality justifies the description of liver cirrhosis as the world's most common immunodeficiency syndrome. Liver cirrhosis is an end-stage organic disease hallmarked by a multifaceted immune dysfunction due to deterioration of antimicrobial recognition and elimination mechanisms in macrophages along with an impaired antigen presentation ability in circulating monocytes. Bacterial translocation supports—and is supported by—uncontrolled activation of immune cell responses and/or loss of toll-like receptor (TLR) tolerance, which can turn exaggerated inflammatory responses to systemic inflammation. Lipopolysaccharide (LPS) or endotoxin boosts systemic inflammatory activity through activation of TLR-2- and TLR-4-dependent pathways and facilitate a massive production of cytokines. This, in turn, results into elevated secretion of reactive oxygen species (ROS), which further enhances intestinal hyperpermeability and thus sustains a vicious circle of events widely known as “leaky gut.” Albumin can be of particular benefit in cirrhotic patients with spontaneous bacterial peritonitis and/or hepatorenal syndrome type of acute kidney injury (HRS-AKI) due to anti-inflammatory and antioxidative stress as well as volume-expanding properties and endothelial-stabilizing attributes. However, presence of autoantibodies against albumin in patients with liver cirrhosis has been described. Although previous research suggested that these antibodies should be regarded as naturally occurring antibodies (NOA), the origin of the antialbumin immune response is obscure. High occurrence of NAO/albumin complexes in patients with liver disease might reflect a limited clearance capacity due to bypassing portal circulation. Moreover, high burden of oxidized albumin is associated with less favorable outcome in patients with liver cirrhosis. To date, there is no data available as to whether oxidized forms of albumin result in neoepitopes recognized by the immune system. Nevertheless, it is reasonable to hypothesize that these alterations may have the potential to induce antialbumin immune responses and thus favor systemic inflammation.

## 1. Liver Cirrhosis-Related Immune Dysfunction

Liver cirrhosis yearly causes 1.2 million deaths worldwide, ranking as the 14th and 10th leading cause of death in the world and in most developed countries, respectively [1]. Overall, almost 35% of cirrhotic patients develop infections of various origins [2]. In the hospital setting, the condition of liver cirrhosis renders patients significantly more susceptible to severe infections [2]. Infection risk is more serious in patients with decompensated cirrhosis than in those with stable liver disease [1]. For example, gastrointestinal hemorrhage—such as from esophageal varices—results in the development of infections in up to 60% of hospitalized patients with underlying liver cirrhosis [3]. Viewed backwards, infections also increase the risk of variceal bleeding [4]. In line with this observation, patients with high serum levels of interleukin-6 (IL-6) and lipopolysaccharide-binding protein (LBP) that were found in association with an impaired intestinal barrier integrity and/or function were also at higher risk for variceal bleeding [5]. In addition, previous prospective studies identified bacterial infections as a predictor for early rebleeding, defined as recurrence of bleeding episodes within one week after admission to the hospital; of those, patients with bacterial infections had a fivefold increased bleeding incidence in comparison to those without infection and a higher 4-week mortality [6]. Finally yet importantly, a prospective study by Goulis et al. confirmed an independent association between bacterial infections and failure to control gastrointestinal hemorrhage in cirrhotic patients [7]. Taken together, infections are the most important precursors of morbidity and mortality as they account for up to 50% of all fatal outcomes in patients with cirrhosis [8]. Hence, increased susceptibility to infections along with a significant risk for infection-related mortality justifies the description of liver cirrhosis as the world's most common immunodeficiency syndrome [9, 10]. Cirrhosis is also reportedly associated with various types of immune dysfunction, which are summarized as cirrhosis-associated immune dysfunction syndrome (CAIDS); for more information on CAIDS, readers are referred to references [10–13].

## 2. Immune Dysfunction and T-Cell Responses

McGovern et al. described a well-known phenomenon in liver cirrhosis: CD4^+^ T-cell deficiency [14]. The authors studied 60 patients with liver cirrhosis; 27 patients suffered from nonviral liver disease, and the remaining 33 patients were diagnosed with chronic hepatitis B or C. The majority of patients showed an abnormal low T-cell count with a mean of 492 CD4^+^ T-cells per *μ*l whole blood. Low CD4^+^ T-cell counts were not significantly associated with the underlying disease (i.e., viral vs. nonviral) but with the presence of splenomegaly. Another study investigated T-cell function in patients with alcoholic liver cirrhosis [15]. *Ex vivo* functional tests such as mitogenic T-cell activation revealed a comparable proliferative response of T-cells. In contrast, intracutaneous tests for common vaccine and environmental antigens (e.g., tetanus toxoid, candida antigen) revealed a hyporesponsiveness of liver patients as compared to healthy controls. In addition, five out of eight patients undergoing vaccination against hepatitis B did not show seroconversion. Thus, next to numerical abnormalities, *in vivo* T-cell function seems to be compromised in patients with severe liver diseases. The exact mechanisms behind this split-tolerance observation remain unknown [16]. Of note, increased numbers of immunoregulatory monocytes and macrophages expressing MER receptor tyrosine kinase (MERTK) have been detected in patients experiencing decompensated cirrhosis and/or acute-on-chronic liver failure; these immune cell phenotypes suppress the innate immune response to microbial agents, and their counts correlate with advanced liver disease and intestinal injury [17].

## 3. Immune Dysfunction and Intestinal Injury

The immunocompromised state of cirrhotic patients reportedly involves loss of the Fc*γ* receptor-mediated elimination pathway against antibody-coated bacteria by macrophages along with an impaired antigen presentation ability resulting from downregulation of human leukocyte antigen DR expression on circulating monocytes (mHLA—DR) [18]. Furthermore, neutrophil cells with degenerated bactericidal skills against *Staphylococcus aureus* or *Escherichia coli* have been reported in association with ethanol-related liver cirrhosis [19]. Thus, liver cirrhosis is an end-stage organic disease characterized by a multifaceted immune dysfunction. This type of immune dysfunction has to be taken into account as an even more serious condition due to the presence of portal hypertension, which ultimately favors extraintestinal spread of gut bacteria [20]. In fact, changes in bacterial translocation behavior along with a gradually attenuated hepatic clearance capacity for antigens—e.g., lipopolysaccharide “LPS” or endotoxin—boost systemic inflammatory activity through activation of various toll-like receptor (TLR) pathways and facilitate a massive production of cytokines [21]. This, in turn, results into elevated secretion of reactive oxygen species (ROS), which further enhance intestinal hyperpermeability and thus sustain a vicious circle of events widely known as “leaky gut” [22, 23]. Meanwhile, a considerable amount of research efforts has been devoted to the development of therapies able to restore and maintain gut barrier integrity. In this regard, evidence for the regulatory potential of the C-X3-C motif chemokine receptor 1 (CX3CR1) in intestinal macrophages has been recently reported [24]. Stepping back through the looking glass, hepatologists are bound to remark that damage of the gut barrier likely promotes bacterial translocation along with systemic inflammation. However, systemic inflammation has further consequences: it aggravates splanchnic vasodilation in the chronic portal-hypertensive state and thus supports ongoing intestinal injury [25]. Interestingly, animal model studies suggest that administration of insulin-like growth factor I (IGF-1) in cirrhotic rats can effectively downregulate the expression of tumor necrosis factor alpha (TNF-*α*) and decisively reduce portal vein pressure, bacterial translocation, and endotoxemia [26]. Of note, valuable insight has been gained in understanding structural alterations of gut barrier integrity due to research that focused on tight junction proteins in patients with liver cirrhosis. Indeed, patients with decompensated cirrhosis had less tight junction protein expression in duodenal biopsy than patients with compensated liver disease [27]. Most intriguing is that other studies enrolling only patients with compensated liver cirrhosis reported downregulation of tight junction proteins in the colon but no significant alteration in tight junction protein expression in gastrointestinal mucosa [28]. This again suggests that functional alterations may equally contribute to derangements of the intestinal permeability.

## 4. Immune Dysfunction and Hepatic Fibrogenesis

Bacterial translocation and intestinal inflammation represent two major promoters of fibrotransformation processes within the liver via the TLR2-dependent pathway [29]. TLR2 interacts with peptidoglycan that is produced by Gram-positive bacteria and thus regulates the number of tumor necrosis factor receptor type I- (TNFRI-) producing TLR2+ monocytes in the lamina propria as well as TNFRI-mediated signals on intestinal epithelial cells [29]. Experiments using transgenic TLR2-/- mice detected bacterial endotoxin, which served as a reliable microbiota translocation marker, at significantly lower levels relative to wild-type mice in systemic blood samples. According to the same authors, decreased gene expression of collagen *α* and decelerated deposition of extracellular matrix proteins render TNFRI-/- mice less vulnerable to liver fibrosis progression [29]. Consequently, the authors conclude that expression of TNFRI on intestinal epithelial cells enhances pathology of the “leaky gut” and markedly induces translocation of bacteria as well as liver fibrogenesis [29]. Furthermore, Seki et al. demonstrated that intestinal bacterial translocation along with a durably activated TLR4 pathway can mediate hepatic fibrogenesis [30]. In agreement with these data, our previous work confirmed the key role of the TLR4-dependent fibrogenesis, especially due to distinct and unfavorable regulation of endogenous TLR4 ligands, such as heat shock protein 8/22 kDa (HSPB8), vs. TLR2-/TLR4- inhibitors, such as inositol polyphosphate 5 phosphatase/145 kDa (INPP5D or SHIP) [21]. Reportedly, cytokines drive chemotaxis in immune cells and facilitate activation of hepatic stellate cells (HSC) at sites of liver inflammation during systemic infections [31]. LPS—a specific TLR4 ligand—triggers chemokine secretion in HSCs and induces chemotaxis of activated Kupffer cells (KC) in vivo [30]. LPS-mediated sensitization of HSCs to transforming growth factor beta (TGF-b) leads to increased expression of fibronectin and collagen and their incorporation into the extracellular in a myeloid differentiation factor 88/nuclear factor kappa B- (MyD88/NF-*κ*B-) dependent manner [30]. Last but not least, uncontrolled activation of immune cell responses and/or loss of TLR tolerance can probably turn exaggerated inflammatory responses to systemic inflammation. Studies on TLR-related genetic variations clearly showed that TLR2 polymorphisms, e.g., the TLR2 GT microsatellite polymorphism and nucleotide-binding oligomerization domain (NOD) 2 variants and/or Arg753Gln (the GA genotype), were associated with an increased risk for spontaneous bacterial peritonitis [32–35]. However, the proposed influence of further polymorphisms, e.g., the TLR4 D299G polymorphism, on the LPS-dependent cytokine response remains controversial. For more information regarding the role of TLR2/TLR4 polymorphisms in liver cirrhosis, readers are referred to references [36–42].

## 5. Immune Dysfunction and Systemic Inflammation

Both bacterial antigens and endogenous molecules expressed upon cell injury, such as pathogen-associated molecular patterns (PAMP) and damage-associated molecular patterns (DAMP), respectively, can trigger systemic inflammation [43]. Interaction between PAMP or DAMP and the innate immune system via specific receptors drives the systemic release of inflammatory mediators [43]. An excessive inflammatory activity seems to play a crucial role also in acute alcoholic hepatitis and/or other settings of acute liver damage [44]. In addition, as mentioned above, a “leaky gut” favoring translocation of bacteria towards the bloodstream along with a gradually impaired hepatic clearance capacity for bacterial antigens may induce activation of TLR pathways and thus further enhance systemic inflammation [21]. The abundantly exacerbated synthesis of cytokines and reactive oxygen species (ROS) driving aggravation of intestinal inflammation and tissue hyperpermeability stresses the importance of establishing therapeutic strategies to overcome them. In this light, it is reasonable to assume that some beneficial effects of albumin administration in cirrhotic patients with spontaneous bacterial peritonitis and/or hepatorenal syndrome type of acute kidney injury (HRS-AKI) might be largely attributable to its anti-inflammatory and antioxidative stress properties [45, 46]. This hypothesis gains considerable support by recent studies in patients with cirrhosis and SBP reporting that combined treatment with intravenous albumin and an antibiotic reduces the risk for renal dysfunction and/or failure and/or mortality in comparison to therapy with a single antibiotic [47]. Albumin is particularly indicated for patients who develop systemic inflammatory response syndrome (SIRS) or sepsis due to its endothelial-stabilizing attributes in addition to its volume-expanding properties [46]. Finally yet importantly, clinical investigations, which clearly identified white blood cell (WBC) count and C-reactive protein (CRP) as independent predictors of in-hospital survival, add great evidence to the prognosis-determining role of systemic inflammation [48, 49]. For more information on immune dysregulation and systemic inflammation in patients with advanced liver diseases, the reader is referred to Lange and Moreau [13].

## 6. Immune Dysfunction and Albumin-Related Immunity

Antialbumin antibodies have repeatedly been described in diseased conditions [50–55] (Table 1). Various groups confirmed the presence of IgG, IgM, and IgA antibodies against albumin in patients with liver disease [56–59]. Hauptman and Tomasi demonstrated an association with hypergammaglobulinemia and hypoalbuminemia [50]. In some reports, an association in particular with viral hepatitis is claimed [60, 61]. Hellstrom et al. showed that albumin-specific T-cells regulate albumin-specific B-cells in patients with chronic hepatitis B (chronic HbsAg carriers) [60]. The authors concluded that—as albumin binds to HbsAg—immunization against albumin is part of the antiviral immune response. However, antibodies with specificity for albumin were also found in patients with nonviral liver disease as published by several other groups [51, 53, 56, 58]. Our own preliminary data on nonviral liver disease indicates the presence of autoantibodies against albumin in patients with liver cirrhosis (unpublished data). The origin of the antialbumin immune response is obscure; Sansonno et al. suggested that these antibodies should be regarded as naturally occurring antibodies (NOA) [62]. NOA are antibodies of IgM or IgG isotype with low affinity and specificity for self-antigens [63]. NOA presumably enhance disposal of “aged” proteins and cells; in addition, an immunoregulatory role seems likely [63]. Thus, NAO against albumin may promote clearance of modified albumin in a physiological manner [63]. Indeed, NOA against albumin have been found in healthy individuals albeit in lower concentration than in patients with liver disease (Table 1). The detection of NOA with specificity for albumin is technically challenging as most of the NOA are bound to albumin in healthy sera [61]. It is essential to separate the antibody/antigen immune complexes to avoid false-negative results. In diseased individuals with liver disease, low albumin levels and hypergammaglobulinemia may result in excessive unbound NAO that can be detected in sera [61]. Interestingly, it has been demonstrated that bypassing portal circulation increases the amount of senescent proteins [64]. The higher occurrence of NAO/albumin complexes in patients with liver disease might therefore reflect an inefficient removal process due to bypassing portal circulation.

**Figure 1:**
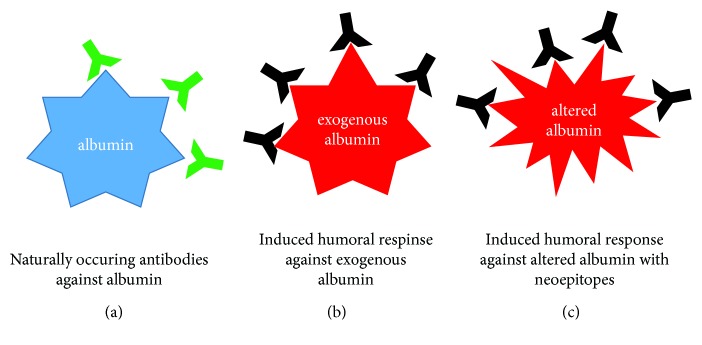
Hypothetical mechanisms promoting antibody formation against albumin. Blue represents the native form of albumin; red represents exogenous albumin or altered forms of native albumin (e.g., oxidized albumin, glycated albumin), both being potentially immunogenic. (a) Naturally occurring antibodies (green Y) may be present in healthy individuals and diseased patients forming immune complexes with albumin. (b) Exogenous albumin may induce a humoral immune response if recognized as foreign antigen. (c) Native albumin might be altered by enzymatic or nonenzymatic processes leading to formation of neoepitopes that are potentially immunogenic. Antialbumin antibodies (black Y) may facilitate the disposal of altered albumin under physiological conditions.

Apart from the concept that antialbumin antibodies are NOA, it is conceivable that treatment with albumin preparations or agents containing albumin may induce formation of an immune response. Genetic variants of albumin have been extensively studied in the past; at least 35 variants have been described so far, and the estimated frequency is about 1 : 3000 [65–71]. However, data on immunogenicity of these variants is rare. Two studies investigated the immunogenic potential of albumin preparations. Brown et al. recruited healthy controls never exposed to exogenous albumin (group I), patients who had either received human serum albumin as part of blood component therapy (group II), and patients who had undergone immunotherapy for allergies for at least one year (group IV) [72]. The agents used for immunotherapy contained human serum albumin in low concentration as stabilizer. The authors did not find increased antialbumin antibody titres in patients that were exposed to exogenous albumin versus healthy, unexposed individuals. However, due to its retrospective design, the study has certain limitations. It is not clearly stated for which disease states and to which extent precisely human albumin preparations were administered in patient group I [72]. Another double-blind, randomized study by Bosse et al. compared the safety of recombinant human albumin with human serum albumin [73]. Two different administration routes were investigated; healthy individuals received either intramuscular injections on five occasions in weekly intervals (*n* = 500 subjects) or intravenous infusions (IV, *n* = 30 subjects). In the IV trial, three doses (10 g, 20 g, and 50 g) were given at three-week intervals. Antibody titres were determined at baseline before first administration and one week after the final dose. Neither intramuscular administration of human serum albumin nor iv administration changed antialbumin antibody titres comparing baseline versus post administration [73]. Therefore, there is currently little evidence that treatment with human serum albumin preparations induces a significant immune response against albumin in healthy individuals. If treatment regimens under diseased conditions may still trigger autoantibody responses against albumin needs to be determined. Furthermore, nonenzymatic and oxidative modifications of albumin have been described to compromise binding, transport, and detoxification capacity of albumin [74–77]. Interestingly, nonenzymatic glycosylation of albumin promotes the formation of potentially immunogenic neoepitopes [54, 78, 79]. In a recent study by Raghav et al., healthy individuals, patients with gestational diabetes mellitus (GD), and patients with type 1 and type 2 diabetes mellitus (T1D, T2D) were recruited [78]. In contrast to healthy individuals, patients with GD, T1D, and T2D showed increased levels of antibodies with specificity for glycated albumin. An earlier study by Mangili et al. reported similar findings; antibodies with specificity for glycosylated albumin were higher in patients with T1D as compared to healthy controls [54]. In patients with coronary artery disease, antibodies against N-homocyteinylated albumin have been detected; the presence of these antibodies was associated with early coronary artery disease [80]. Oettl et al. demonstrated that oxidative modification of albumin is common in patients with liver cirrhosis and even more in patients with acute-on-chronic liver failure [75–77]. High burden of oxidized albumin was associated with less favorable outcome. There is no data available if oxidized forms of albumin result in neoepitopes recognized by the immune system. Nevertheless, it is reasonable to hypothesize that these modifications may have the potential to induce antialbumin immune responses. In summary, humoral antialbumin immune responses have consistently been reported in patients with liver disease. The origin of this immune response is not clear, and several different mechanisms may explain the presence of these antibodies (Figure 1, Table 1).

## 7. Conclusion

Liver cirrhosis is an end-stage organic disease characterized by a multifaceted immune dysfunction. There is evidence for significant dysregulation of the LPS-specific TLR-dependent immunity and the specific T-cell responses that result in aggravated systemic inflammation and high infection-related mortality. Antialbumin immune responses occur in association with liver disease; however, the pathophysiological relevance of this phenomenon remains unclear.

## Figures and Tables

**Table 1:** Key studies on antibodies directed against albumin.

Reference	Patient cohort	Method of detection	Reported result	Comment
Hauptman et al. (1974)	18 patients with Laennec's cirrhosis and history of alcoholic abuse	Affinity purification of cirrhotic sera by adsorption over albumin-loaded column followed immunoelectrophoresis and immunoblot	7 out of 18 patients had detectable IgA antibodies against albumin	Pts. with antialbumin antibodies showed marked hypoalbuminemia and hypergammaglobulinemia

Hellstrom et al. (1989)	8 patients with hepatitis B (all HbsAg^**pos**^, 4 pts. anti-HBe^**pos**^/HBV-DNA^**neg**^)	*Ex vivo* stimulation of isolated B-cells; supernatants were then assayed by ELISA	In all patients, antialbumin IgG was found in the supernatant	

Louzir et al. (1992)	56 patients with HBV-related liver disease (50 pts HBsAg ^pos^, 6 HBsAg^**neg**^, and anti-Hbc^**pos**^)	Patients' sera were measured by ELISA	In 69.3, 64.5, and 24.2% of the pts, antialbumin antibodies of the IgG, IgA, or IgM class were detected	

Lenkei et al. (1980)	275 hepatic patients (“mostly with acute hepatitis”)	Sera were tested by antialbumin (AA) agglutination	51 HbsAg^**neg**^ pts. with high AA-agglutination titre	Antigen: polymerized albumin

Lindstrom et al. (1978)	19 pts, nine pts with prolonged nitrofurantoin therapy and tailing albumin phenomenon (TA), control sera from ten patients without TA	Sera were tested by ELISA	Patients with TA phenomenon showed higher IgG antialbumin levels (measured as absorbance) compared to patients without TA	Follow-up of one patient with TA and nitrofurantoin available: IgG antialbumin levels decreased after cessation of nitrofurantoin

Onica et al. (1983)	8 healthy individuals, 25 patients with various liver disease (14 pts acute viral hepatitis, 8 pts chronic hepatitis, and 3 pts liver cirrhosis)	Affinity purification of sera by adsorption over albumin-loaded column followed radioimmunoassay and immunodiffusion	3 healthy individuals showed antialbumin antibodies (IgG/IgM/no IgA); 10 patients harbored antialbumin antibodies (IgG/IgM/no IgA)	Antigen: polymerized albumin

Tamura et al. (1982)	54 healthy controls, 77 patients with liver disease (8 acute hepatitis, 15 chronic persistent hepatitis, 14 chronic active hepatitis without liver cirrhosis (LC), 16 alcoholic LC, 9 nonalcoholic LC, and 15 hepatocellular carcinoma)	Antibodies in sera or protein fractions were detected with microhaemagglutination assay	Antibodies to human albumin were found in 22% of the patients, antibodies to bovine serum albumin in 48% of the patients	

Brown et al. (1985)	Four groups; group I: 19 healthy individuals who never received immunotherapy or exogenous albumin. Group II: 8 individuals who had received exogenous albumin in the past but no immunotherapy; group III: 26 patients who had received immunotherapy not containing albumin and no exogenous albumin Group IV: 215 patients who had received immunotherapy containing albumin	Antibodies were detected in sera by ELISA	Individuals exposed to albumin did not harbor increased antialbumin titres compared to individuals who were never exposed to albumin	

Bosse et al. (2005)	500 healthy individuals received repeated intramuscular injections in weekly intervals; 30 healthy subjects received intravenous albumin infusions	Antialbumin antibodies were assayed by ELISA from sera	Treatment did not change or increase antibody titres	Double-blind, randomized trial

Mangili et al. (1988)	29 patients with diabetes type 1; 20 healthy individuals	Antialbumin antibodies were assayed by ELISA from sera	Antibodies (IgG/IgM) against modified (glucitol-albumin, ketoamin-albumin) and unmodified albumin were found in both diabetic and healthy individuals; higher titres were more common in diabetic patients	

Raghav et al. (2017)	50 patients with type 1 diabetes, 50 patients with type 2 diabetes, 50 patients with gestational diabetes, 50 patients with type 2 diabetes and chronic kidney disease, and 50 healthy controls	Antialbumin antibodies were assayed by ELISA from sera	Patients with type 1 and type 2 diabetes showed increased levels of antibodies directed against glycated albumin	
